# Experiences of training and implementation of integrated management of childhood illness (IMCI) in South Africa: a qualitative evaluation of the IMCI case management training course

**DOI:** 10.1186/1471-2431-9-62

**Published:** 2009-10-01

**Authors:** Christiane Horwood, Anna Voce, Kerry Vermaak, Nigel Rollins, Shamim Qazi

**Affiliations:** 1 Centre for Rural Health, University of KwaZulu-Natal, Umbilo Road, Durban 4013, South Africa; 2 Department of Public Health, University of KwaZulu-Natal, Umbilo Road, Durban 4013, South Africa; 3 Department of Paediatrics and Child Health, University of KwaZulu-Natal, Umbilo Road, Durban 4013, South Africa; 4 Department of Child and Adolescent Health and Development, World Health Organization, 20 Avenue Appia 1211, Geneva 27, Switzerland

## Abstract

**Background:**

Integrated Management of Childhood Illness (IMCI) is a strategy to reduce mortality and morbidity in children under-5 years by improving management of common illnesses at primary level. IMCI has been shown to improve health worker performance, but constraints have been identified in achieving sufficient coverage to improve child survival, and implementation remains sub-optimal. At the core of the IMCI strategy is a clinical guideline whereby health workers use a series of algorithms to assess and manage a sick child, and give counselling to carers. IMCI is taught using a structured 11-day training course that combines classroom work with clinical practise; a variety of training techniques are used, supported by comprehensive training materials and detailed instructions for facilitators.

**Methods:**

We conducted focus group discussions with IMCI trained health workers to explore their experiences of the methodology and content of the IMCI training course, whether they thought they gained the skills required for implementation, and their experiences of follow-up visits.

**Results:**

Health workers found the training interesting, informative and empowering, and there was consensus that it improved their skills in managing sick children. They appreciated the variety of learning methods employed, and felt that repetition was important to reinforce knowledge and skills. Facilitators were rated highly for their knowledge and commitment, as well as their ability to identify problems and help participants as required. However, health workers felt strongly that the training time was too short to acquire skills in all areas of IMCI. Their increased confidence in managing sick children was identified by health workers as an enabling factor for IMCI implementation in the workplace, but additional time required for IMCI consultations was expressed as a major barrier. Although follow-up visits were described as very helpful, these were often delayed and there was no ongoing clinical supervision.

**Conclusion:**

The IMCI training course was reported to be an effective method of acquiring skills, but more time is required, either during the course, or with follow-up, to improve IMCI implementation. Innovative solutions may be required to ensure that adequate skills are acquired and maintained.

## Background

Infant and child mortality remains high in developing countries, where almost 10 million deaths occur annually in children under-5 years old [1]; most deaths are from common, preventable and easily treatable childhood diseases [2]. The millennium development goal for child mortality commits nations to reduce child deaths by two thirds by 2015 [3]. However, in South Africa, child mortality has risen over the past decade. HIV/AIDS is the commonest cause of death in children under-5 years, but diarrhoeal disease, pneumonia and malnutrition remain important causes of mortality [4]. More than 60% of global child deaths could be prevented by proven interventions available and affordable today [5], but coverage remains low particularly in low income countries [6]. The challenge is to improve coverage of child survival interventions to a level that will have a positive impact on child mortality [7].

Integrated Management of Childhood Illness (IMCI) is a strategy developed by the World Health Organisation (WHO) and United Nations Children's Fund (UNICEF) to improve child survival in resource poor settings [8]. IMCI seeks to improve case management skills of first level health workers, strengthen the health system for effective management of sick children, and promote good family and community child care practices [9]. The child is treated holistically with evidence based interventions that are feasible to implement in countries where most child deaths occur [5]. South Africa adopted IMCI in 1997, and is one of over 100 developing countries to do so [10].

At the core of the IMCI strategy are integrated guidelines for assessment and management of sick children at primary level, focussing on the conditions that cause most child deaths [11]. Rather than make a diagnosis, IMCI practitioners classify the child's illness according to severity using a series of algorithms, from which specific treatments are identified. IMCI guidelines are built around a series of simple questions, and easily recognised and defined signs and symptoms [12-14], with emphasis on nutrition, health promotion and counselling. Primary care workers are trained in IMCI using a structured training course developed by WHO, which is supported with extensive learning materials. Course participants receive a chart booklet containing all the IMCI guidelines to use as a desk reference. The 11 days of training combines classroom work with hands-on clinical practise, and participants achieve competency by repetition, combined with individual feedback from facilitators [15]. To achieve high quality training, IMCI facilitators are carefully selected, on the basis of their performance, to attend an additional 5-day IMCI facilitators training course. WHO recommends at least one facilitator for every four participants, and tools are provided for intensive monitoring of participants' progress. A course director oversees the quality of the training, and reviews the performance of participants [15]. A detailed guide directs facilitators in conducting each learning activity, so that content and activities are largely consistent between different training sites and different countries. All IMCI trained health workers should receive at least one follow-up visit in their own health facility after training, to reinforce their skills and solve implementation problems [16].

IMCI provides a model for comprehensive implementation of proven public health interventions. An evaluation carried out in 5 countries [17] showed improvements in health worker performance following IMCI training [18,19]. Children seen by IMCI trained health workers were significantly more likely to receive correct treatments, and IMCI trained health workers communicated better with carers [20]. Although IMCI consultations take longer, IMCI was shown to be efficient [21] and cost less than routine care in some settings [22]. Despite these improvements, absolute levels of health worker performance were often poor. In Uganda, less than half of children received correct treatment [23], and in Peru this was as low as 10% [24]. Even in the most successful implementation sites there was considerable room for improvement [25].

If child survival is to be improved, reasons for poor performance of IMCI trained health workers must be identified and understood. The knowledge and skills acquired during training are important determinants of performance, but performance is also influenced by other factors, including health workers' perceptions and motivation, attitudes of the client and community, and the environment in the health facility. Health workers face continually changing environments, so even if a new guideline is fully understood they may not replace their pre-existing practise, but are more likely to modify it to incorporate some aspects of the new guidelines [26]. Supervision has been shown to improve performance [27] and can bridge the gap between knowledge and practise. It is, therefore, important to know how health workers experience learning and implementing IMCI, and their experiences of follow-up visits, to understand what determines health workers' ability to acquire new skills and transfer them to the workplace.

There have been no previous published evaluations of IMCI case management training since its initial field test in 1997[28]. In this article we describe the experiences of the content and methodology of the IMCI training from the perspective of IMCI trained health workers in South Africa, and whether health workers feel that they were given the skills to implement IMCI. We also investigate their experiences of IMCI implementation, including barriers and enabling factors to implementing IMCI in the workplace, and experiences of follow-up after training.

## Methods

### Setting

Focus group discussions (FGD's) with IMCI trained health workers were conducted in two provinces of South Africa, KwaZulu-Natal (KZN) and Limpopo, in April and May 2006. Both provinces began implementing IMCI in 1998 and, at the time of our study, 1325 health workers had been trained in Limpopo and 1300 in KZN, comprising 47% and 32% of health workers seeing children in primary health care (PHC) clinics respectively. IMCI was being implemented in 283 of 474 (60%) PHC clinics in Limpopo, and 387 of 604 (64%) clinics in KZN. All IMCI trained health workers working in PHC clinics are registered nurses. A total of five FGD's were conducted, three in KZN and two in Limpopo. Ethical approval was obtained from the Biomedical Research Ethics Committees of the University of KwaZulu-Natal Medical School, Durban, and WHO, Geneva.

### Respondents

Limpopo and KZN provinces were selected at the request of the Department of Health, because they are regarded as the two provinces at the forefront of IMCI implementation in South Africa. Urban and rural districts were then selected in each province based on convenience, three districts in KZN, and two in Limpopo. IMCI co-ordinators from each district selected up to 10 IMCI trained nurses to participate in the focus groups. Nurses were purposively selected on the basis that they had attended an 11-day IMCI course, were currently working in a PHC clinic, and that they would contribute to a discussion of IMCI implementation. These selection criteria were applied to ensure that respondents had relevant experience of IMCI training and implementation, and would provide in-depth information for the discussions. This approach is inherent in purposive sampling strategies, where the focus is on understanding important cases rather than on generalising from a sample to a population[29].

Respondents were informed in writing about the research and invited to participate. They were compensated for transport costs and received refreshments, but there was no other incentive to participate. Written consent was obtained, and anyone not wishing to participate was free to refuse. Respondents were assured that individual comments would remain confidential.

Discussions with nurses were conducted by an experienced researcher (AV) with the help of an observer who took notes (CH). Discussions were conducted in English and were audio recorded. The discussions were open-ended, and allowed respondents to direct the discussion.

### Analysis of the data

The audio tapes were transcribed verbatim. The transcriptions, together with the observation notes, were analyzed by two researchers independently. Major and minor themes were identified through manual content analysis of the transcripts, and differing interpretations were discussed in order to reach consensus. Consensus was reached on all themes.

## Results

### General experiences

Overall, respondents found the IMCI training interesting, informative, empowering, and transformative. There was consensus that the training improved their skills and confidence in managing sick children. Respondents were relieved that they would not be formally assessed, and they found the learning situation relaxed, and accepting.

"It was eye-opening, very much interesting, but phew the workload! But at the end it's just that it's nice, because we are not going to write a test, you grasp whatever you can." (FGD 4)

"The situation was relaxed, there is no shouting ... and there is no shame if you are left behind. No, you just feel the same like others." (FGD 1)

Respondents experienced the facilitators as dedicated and friendly. Facilitators took time to explain, were knowledgeable, well-informed and experienced, and had a lot of understanding of the context within which the health workers functioned.

" [the facilitators] convey the message alright, and they are kind and friendly, they don't have attitudes, the situation was relaxed. They seem to have a lot of understanding, a lot of knowledge, because if you ask some questions, they just answer you there and then. Yes, and they can spot a person who is behind, and they can just assist you." (FGD 1)

"They [facilitators] were people who had worked in the clinics before, so they knew what problems we go through, making it easier." (FGD 4)

During IMCI training, progress of participants' is monitored and additional help provided as necessary. It was frequently mentioned that facilitators were responsive to '*slow learners*', so it appeared that respondents noticed and appreciated this.

"When we go to the clinical session- let's say that person has missed a sign, then they [facilitators] will be with this person and show her how to identify a positive sign on that child, so that she can see her mistake and next time she will be able to do it correctly." (FGD 3)

### Content of the training

The content of IMCI training was described as comprehensive, covering all aspects of managing a sick child in a way that simplified the management of childhood illnesses, and reduced the anxiety previously felt about assessing and caring for children, especially young babies.

"For me it was exciting, because before I used to have an attitude about assessing a child. I wasn't sure what to look at in a child, especially a newborn, it was not easy for me. But after IMCI I gained a lot of skills and knowledge, and now for me it is easy when I am going to assess a child, there's not much problem." (FGD 4)

In particular respondents mentioned that they found the HIV component of IMCI to be "*very good" *and *"very important"*, particularly when dealing with the mother of a child suspected to be infected with HIV.

"It gave me the skills .. on how to approach the mother [about HIV], which was a bit difficult before, but after doing IMCI I started enjoying it ... I even developed my own tricky ways of approaching the mother." (FGD 1)

However, many respondents felt there was too much information to learn in 11 days of training. The first week is spent on the module 'the sick child aged 2 months up to 5 years', and 'the sick young infant aged 1 week up to 2 months' is completed during the second week, together with modules on counselling and follow-up for sick children. There was consensus that more time was needed for the sick young infant, which was felt to be very important. Similarly, some respondents felt that the time allocated to breastfeeding was inadequate.

"Eleven days is too strenuous. By the time when we come to the end of the course everybody is just exhausted, they don't absorb anything, especially with the sick young infant .... At least the first part of it, the first week, it's good." (FGD 1)

"When coming to breastfeeding, I gained the experience after training, not during training. The facilitators were very much fast, they were in a hurry." (FGD 2)

### Training Methods: Classroom

Training methods are highly structured, and a variety of teaching methods are used. Participants are given reading, written exercises, and case studies to complete, and feedback is given to individuals, or to the whole group. There are presentations from facilitators, group discussions, role-plays, photographs, and video presentations. There was general agreement that the training methods were good, helpful, improved understanding of the course content, and facilitated integration between theory and practice. Respondents appreciated the variety of adult learning methods employed, and the reinforcement that was provided by utilising different methods to cover the same material. They also appreciated the *"recapping" *sessions at the beginning of each day.

"The methods [were] very good, [they] involved all the types of methods used in teaching. A theoretical and a practical [part], and the exercises in between so we can practise what we learnt, the demonstrations, the role plays and also the exercises. And there is a lot of repetition, that's why at the end of the training they are now able to implement what they have learnt, because they repeat." (FGD 3)

Some respondents expressed that the course materials were user-friendly and easy to read.

"The chart booklet as well as the manuals they are user-friendly, it is easy to follow them, they are short, straight to the point and you know it's easy to read" (FGD 1)

### Training methods: Practical

Participants attend one-hour clinical sessions in the hospital paediatric ward, and in the PHC clinic each day. Clinical teaching is provided by the facilitators in the clinic, and by a doctor on the ward who is trained in IMCI clinical instruction. Respondents stated that the practical component was useful to reinforce the theory they had learnt, and to learn clinical skills. A major theme identified was that respondents felt they needed more time for the practical, particularly because of the travelling involved.

"During practical there are doctors who guide us. They tell you 'go and listen to that child and hear the sound', and they will tell you 'this is the sound', and then if you don't know that sound, you listen to the child ... oh, that is the one they are talking about ... [We know] because we have experienced it in the practical" (FGD4)

"The practical part is not enough, because you have to be in the ward maybe [for] plus or minus one hour, doing three or four cases, and then after that you go back to the clinic so you had to rush." (FGD 1)

### Training period

IMCI training is conducted for 11 days over two consecutive weeks. It was strongly and repeatedly expressed by most respondents that the period of training was too short. However, in one focus group, some did feel that the period of training "*was OK*".

"The training period was too short for what we've been doing. It was too short, and there were more modules to read at the same time... I think maybe if you can just add some other weeks to the training and just give it some [more] time." (FGD 1)

"The period of training, I think it was OK, because the participants had that time of being in the classroom where they go through all the manuals, and then they also have a session of going out to the clinical session where they implement what they have learnt in the class." (FGD 3)

### Follow up after training

Follow-up visits are considered part of IMCI training, and should be carried out 4-6 weeks after IMCI training to help new IMCI practitioners transfer their skills to the workplace. Some respondents reported that it took months, sometimes years, before they received a follow-up visit. Respondents anticipated the follow-up visit with ambivalent feelings: they wanted the visit, but were unsure how their performance would be rated. Most respondents reported either no follow-up or one follow-up visit, but some reported receiving a second visit during a provincial IMCI review. No one reported receiving ongoing follow-up. Experiences of follow-up visits were generally positive: during the visit facilitators helped to affirm correct practice, and provided guidance if modifications were required. Follow-up visits motivated newly trained IMCI practitioners and helped gain the support of non-IMCI trained staff.

"They always tell us that they will do follow-up in 4 to 6 weeks, but it takes long, even two years or so.... At the clinic I was the first one to be trained in IMCI and I was stranded alone without having their [the facilitators'] support. I tried to update my colleagues, but they were reluctant to change."FGD2)

"On my side, after a few months, someone came from the district office to check if I had some problems .... At first there was some shivering [apprehension]. We sat down and we talked, after we talked about some conditions I found that I was on the right track."(FGD 2)

" [after the follow-up visit] we have that motivation ... [and] the support from our colleagues, ... it even made other people in the clinic want to go on the training"(FGD 3)

### IMCI implementation: enabling factors

Respondents in all focus groups consistently expressed that their confidence in managing children, particularly young infants, had increased, and that they felt empowered and knowledgeable in their practise after attending the course.

"It was quite an exciting experience. Before it wasn't nice; I didn't like to examine a child because I didn't have the skills to do that. But after IMCI in fact it was quite a nice experience ... so really it opened my eyes." (FGD 4)

" You work with confidence because you know what you are doing. Like before I used to go to the place where there were no children because I really was not too sure what I was doing, but since I know this IMCI I know exactly what I am doing." (FGD 1)

Implementation of IMCI was consistently described by respondents as being time consuming, but this was also seen as beneficial in improving the relationship with mothers, and the respondents' confidence improved when using the IMCI approach led to the mothers seeing them with a new respect.

"In IMCI we ought to examine the child from head to toe, so most of the mothers they enjoy the examination, so they encourage others in the community." (FGD3)

The chart booklet was seen as a useful desk aid, which guided them in the management of children and improved their confidence in transferring their skills to the workplace after training.

"And then if you are assisting a child, you open your IMCI book, so you can no longer make a mistake, that's what's nice because you are not alone, everything you've got there." (FGD 4)

"Now I know the right dose [of medication] that must be given. So after IMCI, whether you are a genius or not, the chart booklet must be there all the time." (FGD 2)

Other factors mentioned as facilitating implementation of IMCI was the support of colleagues, especially those who were IMCI trained, and that treatments recommended by IMCI are available at the clinic.

"We have that motivation from the people that were trained before you, the support from our colleagues" (FGD3)

"One thing I can say is the treatment, because we have got it at the clinic, so if the chart booklet says 'give this for that' you know that you [have] got it." (FGD4)

### IMCI implementation: barriers

The biggest barrier to IMCI implementation, that was consistently mentioned, was that IMCI consultations take longer, which is a particular problem given staff shortages in many clinics.

"I am the only one who is IMCI trained, so it is not easy to do it the correct way because I will not be able to finish these children by 4 pm." (FGD4)

Lack of support from colleagues in the clinic, particularly those not trained in IMCI, was identified as a major barrier. Encouraging other members of the health team, including ambulance services and doctors at the referral hospital, to support IMCI was expressed as a challenge.

"The people who are not [IMCI] trained, they seem to have a negative attitude towards the people who are IMCI trained, because that person will not understand why is it that you are taking too long to finish your clients."(FGD 4)

"If the sister in charge of the clinic did not undergo this training she will not understand the IMCI language and if I want to implement IMCI it will be a problem." (FGD2)

The time taken for IMCI consultations did cause longer waiting times. Respondents described dealing with this situation in several different ways; non-IMCI trained nurses took over care of some children, IMCI was only partially implemented, or some children did not get seen at the end of the day.

" Sometimes you find that someone who is not trained is [seeing children] ...other nurses are just outside helping to consult." (FGD 3)

"In other clients, you can see, you cannot give one hundred percent what you are supposed to give, because you have to push the line [reduce the queue]." (FGD 1)

"You have to cut the patients when it is 4 o'clock. So you see, you won't be able to see them all. We will implement [IMCI], but we won't manage to see them." (FGD3)

Patient expectations were also identified as a barrier; mothers expect to receive treatments that are no longer used according to IMCI. This is worse if the practises of IMCI trained and non-IMCI trained nurses are inconsistent.

"They [mothers] are unhappy because we are not all trained. So on another day they will see someone who will give paracetomol when the temperature is 36, and they get confused." (FGD2)

"They are expecting to go home with something. We tell them to give the hydration at home, or the cough remedy at home, they don't like it." (FGD4)

### Recommendations suggested by respondents

Respondents suggested that all clinic nurses be trained in IMCI, and that all IMCI trained nurses also be trained in prevention of mother to child transmission of HIV (PMTCT) and HIV/AIDS counselling. In addition, they felt that the period of IMCI training should be increased, with more emphasis on the module for the sick young infant. Suggestions included that the training be split into two separate weeks, or that an extra week be allocated to the sick young infant. Respondents felt that clinical sessions could be re-organised, to reduce the time spent travelling for short clinical sessions. They also suggested that increasing IMCI coverage would strengthen implementation, that facilitators work full-time on IMCI, and that more follow-up visits and IMCI update workshops should be held.

"You would start with the sick child from [age] two months to five years, let's say [this is covered over] two weeks and then another week [for the sick young infant]... because now we do a lot of travelling ... that was really exhausting, plus homework." (FGD 1)

## Discussion

Overall, the IMCI training course was consistently very well received by respondents, who reported that the variety of methodologies enhanced the experience of the training, and reinforced the skills and knowledge required. Training materials were well understood by participants. The WHO approach of providing detailed guidelines for facilitators supported by complex, well-developed training materials ensures that training is interesting, well prepared and information is consistent. It requires skills and resources to develop high quality training materials, so providing generic materials which can then be adapted at country level [30]worked very well in our setting.

IMCI facilitators are carefully selected, well trained, and supervised by a course director, and the high ratio of facilitators to participants allows careful monitoring of participants' progress. In this evaluation, facilitators were consistently rated very highly by respondents in terms of their knowledge and exceptional commitment, as well as for their ability to identify problems and help individual participants. From this evaluation it can be said that strict selection criteria for facilitators helped to ensure good quality of IMCI training, which has been maintained during the expansion of the IMCI strategy.

Health workers in all groups strongly endorsed the IMCI approach to assessment and management of a sick child, frequently saying that this had been a challenging area of their practise prior to IMCI training, and that they gained confidence and skills from IMCI. The training was felt to be comprehensive, and participants gained most of the required skills. The approach of using simple, defined signs and symptoms to assess a child gave practitioners clarity and confidence in their practise.

The major problem identified with IMCI training was a lack of sufficient time during the course. One reason for this is that, in South Africa and other high HIV prevalence countries, an HIV component has been added to IMCI without extending the duration of the course [31]. Although respondents attended IMCI courses in different districts and at different times, in all but one of the FGD's, lack of time was the strongest theme in all areas of the discussion. It was felt that time was too short for completion of all activities, that the workload was excessive, and too much time was spent travelling, making the clinical sessions rushed. In particular, participants did not get maximum benefit from the modules on the young infant and breastfeeding. Although respondents mentioned that there was a lot of repetition, this was felt to be needed for them to learn the materials thoroughly. Given shortages of skilled staff in many developing countries, it is difficult for health workers to leave their workplace for 11 days of training, and conducting an IMCI course is logistically difficult and expensive. This makes it difficult to extend the training period, and in several countries the training course has been shortened [32], although this may adversely affect performance[23]. If benefits to child survival are expected from IMCI, given the poor performance of IMCI trained health workers[33], is it realistic to expect that all skills required for management of sick children can be acquired over 11 days, and that these skills can be maintained for years to come?

If longer training is not feasible, alternative approaches could be taken. The course could be split into two week-long components, giving participants time to practise their skills before moving to the second week. Course participants could be given self-learning tasks away from the classroom, for example video or written exercises, or a case log, which could be assessed by facilitators. Interactive computer-based learning methods could also be a useful aid to learning. Clinical practise could be re-organised, with fewer longer clinical sessions, and clinical practise away from the course could be introduced. A formal assessment on completion of training could replace some of the monitoring currently undertaken during the course, despite respondents in this study saying that the lack of a formal assessment was a positive aspect of the training. As health worker performance has been shown to be weakest in assessment of severely ill children [33,34], a formal assessment would also ensure that all IMCI practitioners reach competency in assessing those signs on which these classifications depend. Similar strategies could be used to maintain IMCI skills over time.

Training alone has been shown to have little lasting effect on health worker performance [35], and adherence to guidelines may be poor regardless of having undergone training [36]. So, for sustainable improvements, training should be combined with other approaches, including supervision [37]. IMCI requires radical changes in practise, so follow-up visits are critical for helping newly trained IMCI practitioners to transfer their skills to the workplace. Although respondents reported that follow-up visits were beneficial, these were frequently delayed, which can lead to a loss of skills [27]. Follow-up visits include assessment of clinical competence, so IMCI supervisors must be competent IMCI practitioners, and should attend an additional 5-day course on IMCI follow-up. In KZN and Limpopo IMCI facilitators do follow-up visits because clinic supervisors do not have the skills, making clinical supervision unsustainable. Implementing and sustaining follow up after IMCI training has repeatedly been shown to be a problem in Bangladesh, Tanzania, Peru and Uganda [19,23-25], suggesting that follow-up should be reviewed to make it less resource intensive. For example, newly trained IMCI practitioners could come to a central clinical venue where observations and discussions could be used to provide support for implementation. Ongoing IMCI updates, as suggested by respondents, could give opportunities for IMCI practitioners to meet, solve problems together, and provide peer support. Other methods of improving implementation like awarding clinics 'IMCI excellent' accreditation could be used to motivate practitioners. A recent study evaluating interventions to improve performance of IMCI trained health workers, showed that a combination of additional supports following training (training and support for supervisors, ongoing supervision, additional job aids, and non financial incentives) improved performance of health workers significantly. This suggests that success in maintaining health worker performance requires interventions that target multiple determinants of health worker practise, although providing ongoing supervision remained a problem [38].

The additional time taken for an IMCI consultation was expressed as a barrier to implementation, leading to partial implementation of IMCI, which has been repeatedly shown in assessments of IMCI implementation [23]. Counselling messages recommended by IMCI are time consuming for health workers to deliver, and health workers under time pressure could limit counselling messages to those that are most essential, or alternative ways of delivering counselling messages, for example written materials, could shorten the consultation time. However, IMCI skills improve with practise [39], and newly trained IMCI practitioners need support in the initial period after completion of training if they are to gain confidence. Time pressures could be reduced by allocating a reduced workload initially, and other nurses and child carers could be informed that IMCI implementation may take longer initially, but this is due to improvements in the care of sick children. Another important barrier was carers demanding medication not recommended by IMCI, which has also been a problem in other settings [37]. If treatments are to be discontinued, this policy should be implemented consistently and guidelines provided for all health workers seeing children, or if possible, such medications could be removed from the clinic. If not addressed, this issue will continue to undermine IMCI implementation.

Limitations to this study include that respondents for the focus group discussions were selected by IMCI co-ordinators who were familiar with respondents and may have been involved in their training. Co-ordinators however, were not present at FGD's, and although they knew IMCI was to be discussed they were not told that the training specifically was being assessed. Respondents were told that individual comments would be confidential, but it is possible that they had the perception that researchers wanted to hear positive things about IMCI training and implementation.

## Conclusion

IMCI training course participants gave positive feedback about the content and methodology of the IMCI training, however it was strongly felt that the course was too short to acquire all skills and follow-up after training is currently insufficient to bridge this gap. Further research is required to prospectively assess the training and determine whether different training approaches could improve health worker performance. If IMCI coverage is to be improved sufficiently to have a beneficial effect on child survival, innovative solutions are urgently required to ensure that newly trained practitioners can transfer their skills to the workplace and maintain these skills over time. These approaches would also need further research to determine which interventions would be most effective.

## Competing interests

The authors declare that they have no competing interests.

## Authors' contributions

CH was the principal investigator for the study, designed the study, supervised data collection, analysed the data and wrote the paper. AV advised on data collection, conducted the focus groups, analysed the data and helped write the paper. KV participated in designing the study, analysing the data and writing the paper. NR and SQ advised on the design of the study, the analysis of the findings and the writing of the paper. All authors read and approved the final manuscript.

## Pre-publication history

The pre-publication history for this paper can be accessed here:

http://www.biomedcentral.com/1471-2431/9/62/prepub

## References

[B1] UNICEF The state of the worlds children 2008: child survival 2007 New York: UNICEF http://www.unicef.org/sowc08/report/report.php

[B2] Black RE Morris SS Bryce J Where and why are 10 million children dying every year? Lancet 2003 9 9376 2226 2234 10.1016/S0140-6736(03)13779-8 12842379

[B3] UN Rights United Nations Millenium Declaration 2000 http://www.un.org/millennium/declaration/ares552e.htm

[B4] Bradshaw DBD Nannan N What are the leading causes of death among South African Children? MRC Policy Brief 2003 9

[B5] Jones G Steketee RW Black RE Bhutta ZA Morris SS How many child deaths can we prevent this year? Lancet 2003 9 9377 65 71 10.1016/S0140-6736(03)13811-1 12853204

[B6] Bryce J El Arifeen S Pariyo G Lanata C Gwatkin D Habicht JP Reducing child mortality: can public health deliver? Lancet 2003 9 9378 159 164 10.1016/S0140-6736(03)13870-6 12867119

[B7] Victora CG Hanson K Bryce J Vaughan JP Achieving universal coverage with health interventions Lancet 2004 9 9444 1541 1548 10.1016/S0140-6736(04)17279-6 15500901

[B8] Tulloch J Integrated approach to child health in developing countries Lancet 1999 9 Suppl 2 SII16 SII20 10.1016/S0140-6736(99)90252-0 10507254

[B9] Bryce J Victora CG Habicht JP Black RE Scherpbier RW Programmatic pathways to child survival: results of a multi-country evaluation of Integrated Management of Childhood Illness Health policy and planning 2005 9 Suppl 1 i5 i17 10.1093/heapol/czi055 16306070

[B10] WHO Child and adolescent health and development progress report 2002-2003 Geneva 2004 63 66

[B11] Gove S Integrated management of childhood illness by outpatient health workers: technical basis and overview. The WHO Working Group on Guidelines for Integrated Management of the Sick Child Bulletin of the World Health Organization 1997 9 Suppl 1 7 24 9529714 PMC2486995

[B12] Kalter HD Burnham G Kolstad PR Hossain M Schillinger JA Khan NZ Saha S de WV Kenya-Mugisha N Schwartz B Evaluation of clinical signs to diagnose anaemia in Uganda and Bangladesh, in areas with and without malaria Bull World Health Organ 1997 9 Suppl 1 103 111 9529723 PMC2486990

[B13] Weber MW Kellingray SD Palmer A Jaffar S Mulholland EK Greenwood BM Pallor as a clinical sign of severe anaemia in children: an investigation in the Gambia Bulletin of the World Health Organization 1997 9 Suppl 1 113 118 9529724 PMC2487006

[B14] Zucker JR Perkins BA Jafari H Otieno J Obonyo C Campbell CC Clinical signs for the recognition of children with moderate or severe anaemia in western Kenya Bull World Health Organ 1997 9 Suppl 1 97 102 9529722 PMC2486997

[B15] World Health Organization Integrated management of childhood illness: conclusions. WHO Division of Child Health and Development Bulletin of the World Health Organization 1997 9 Suppl 1 119 128 9529725 PMC2486994

[B16] Lambrechts T Bryce J Orinda V Integrated management of childhood illness: a summary of first experiences Bulletin of the World Health Organization 1999 9 7 582 594 10444882 PMC2557705

[B17] Bryce J Victora CG Habicht JP Vaughan JP Black RE The multi-country evaluation of the integrated management of childhood illness strategy: lessons for the evaluation of public health interventions American journal of public health 2004 9 3 406 415 10.2105/AJPH.94.3.406 14998804 PMC1448266

[B18] Armstrong Schellenberg J Bryce J de Savigny D Lambrechts T Mbuya C Mgalula L Wilczynska K The effect of Integrated Management of Childhood Illness on observed quality of care of under-fives in rural Tanzania Health policy and planning 2004 9 1 1 10 10.1093/heapol/czh001 14679280

[B19] El Arifeen S Blum LS Hoque DM Chowdhury EK Khan R Black RE Victora CG Bryce J Integrated Management of Childhood Illness (IMCI) in Bangladesh: early findings from a cluster-randomised study Lancet 2004 9 9445 1595 1602 10.1016/S0140-6736(04)17312-1 15519629

[B20] Gouws e Bryce J Habicht JP Amaral J Pariyo G Schellenberg JA Fontaine O Improving antimicrobial use among health workers in first-level facilities: results from the multi-country evaluation of the Integrated Management of Childhood Illness strategy Bull World Health Organ 2004 9 7 509 515 15508195 PMC2622903

[B21] Bryce J Gouws E Adam T Black RE Schellenberg JA Manzi F Victora CG Habicht JP Improving quality and efficiency of facility-based child health care through Integrated Management of Childhood Illness in Tanzania Health policy and planning 2005 9 Suppl 1 i69 i76 10.1093/heapol/czi053 16306072

[B22] Adam T Manzi F Schellenberg JA Mgalula L de Savigny D Evans DB Does the Integrated Management of Childhood Illness cost more than routine care? Results from the United Republic of Tanzania Bulletin of the World Health Organization 2005 9 5 369 377 15976878 PMC2626239

[B23] Pariyo GW Gouws E Bryce J Burnham G Improving facility-based care for sick children in Uganda: training is not enough Health policy and planning 2005 9 Suppl 1 i58 i68 10.1093/heapol/czi051 16306071

[B24] Huicho L Davila M Campos M Drasbek C Bryce J Victora CG Scaling up integrated management of childhood illness to the national level: achievements and challenges in Peru Health policy and planning 2005 9 1 14 24 10.1093/heapol/czi002 15689426

[B25] Armstrong Schellenberg JR Adam T Mshinda H Masanja H Kabadi G Mukasa O John T Charles S Nathan R Wilczynska K Effectiveness and cost of facility-based Integrated Management of Childhood Illness (IMCI) in Tanzania Lancet 2004 9 9445 1583 1594 10.1016/S0140-6736(04)17311-X 15519628

[B26] Rowe AK de Savigny D Lanata CF Victora CG How can we achieve and maintain high-quality performance of health workers in low-resource settings? Lancet 2005 9 9490 1026 1035 10.1016/S0140-6736(05)67028-6 16168785

[B27] Chaudhary N Mohanty PN Sharma M Integrated management of childhood illness (IMCI) follow-up of basic health workers Indian J Pediatr 2005 9 9 735 739 10.1007/BF02734143 16186673

[B28] World Health Organization Integrated management of childhood illness: field test of the WHO/UNICEF training course in Arusha, United Republic of Tanzania. WHO Division of Child Health and Development & WHO Regional Office for Africa Bull World Health Organ. 1997 9 Suppl 1 55 64 9529718 PMC2486999

[B29] Patton MQ Enhancing the quality and credibility of qualitative analysis Health Serv Res 1999 9 5 Pt 2 1189 1208 10591279 PMC1089059

[B30] World Health Organization IMCI Adaptation Guide Geneva 2002

[B31] Qazi SA Muhe LM Integrating HIV management for children into the Integrated Management of Childhood Illness guidelines Trans R Soc Trop Med Hyg. 2006 9 1 10 13 16257023 10.1016/j.trstmh.2005.05.013

[B32] World Health Organization The analytic review of the integrated management of childhood illness strategy Geneva 2003 26

[B33] Horwood C Vermaak K Rollins N Haskins L Nkosi P Qazi S An evaluation of the quality of IMCI assessments among IMCI trained health workers in South Africa PLoS One 2009 9 6 e5937 10.1371/journal.pone.0005937 19536288 PMC2693922

[B34] Health worker performance after training in integrated management of childhood illness--Western Province, Kenya, 1996-1997 MMWR Morb Mortal Wkly Rep 1998 9 46 998 1001 9843326

[B35] Haines A Kuruvilla S Borchert M Bridging the implementation gap between knowledge and action for health Bulletin of the World Health Organization 2004 9 10 724 731 discussion 732 15643791 PMC2623035

[B36] Rowe AK Hamel MJ Flanders WD Doutizanga R Ndoyo J Deming MS Predictors of correct treatment of children with fever seen at outpatient health facilities in the Central African Republic Am J Epidemiol 2000 9 10 1029 1035 10853642 10.1093/oxfordjournals.aje.a010131

[B37] Rowe AK Onikpo F Lama M Cokou F Deming MS Management of childhood illness at health facilities in Benin: problems and their causes American journal of public health 2001 9 10 1625 1635 10.2105/AJPH.91.10.1625 11574325 PMC1446844

[B38] Rowe AK Onikpo F Lama M Osterholt DM Rowe SY Deming MS A multifaceted intervention to improve health worker adherence to integrated management of childhood illness guidelines in Benin American journal of public health 2009 9 5 837 846 10.2105/AJPH.2008.134411 19299681 PMC2667861

[B39] Kelly JM Rowe AK Onikpo F Lama M Cokouits F Deming MS Care takers' recall of Integrated Management of Childhood Illness counselling messages in Benin Trop Doct 2007 9 2 75 79 10.1258/004947507780609257 17540083

